# Knowledge, Attitudes, Prevalence and Associated Factors of Cigarette Smoking Among University Students: A Cross Sectional Study

**DOI:** 10.1007/s10900-020-00874-0

**Published:** 2020-07-06

**Authors:** Omar Al Omari, Loai Abu Sharour, Karen Heslop, Dianne Wynaden, Abdullah Alkhawaldeh, Mohammad Al Qadire, Atika Khalaf

**Affiliations:** 1 College of Nursing, Sultan Qaboos University, 123 Al-Khoud, Muscat, Oman; 2 College of Nursing, ALZaytoonah University of Jordan, Amman, 11733 Jordan; 3 School of Nursing, Midwifery and Paramedicine, Curtin University, Perth, WA U1987 Australia; 4 College of Nursing, Jerash University, Jerash, 26150 Jordan; 5 College of Nursing, Al Al-Bayt University, Mafraq, 25113 Jordan; 6 Faculty of Health Science, Kristianstad University, 291 88 Kristianstad, Sweden

**Keywords:** Smoking habits, Gender-specific approach, Preventive actions, University role

## Abstract

This study aimed to assess the prevalence of smoking and associated sociodemographic and economic factors as well as students’ knowledge about and attitudes towards smoking among university students in Oman. A proportionate random sampling technique recruited 401 students from three universities in a cross-sectional study. The prevalence of smoking was 9.0%. Significant differences in gender, place of residence, if participants had received medical advice, years spent at the university, student income/day, family members who smoked, knowledge and attitude scores were identified. Universities in collaboration with health care providers should be leading the development of strategies to reduce the prevalence of smoking and to sustain the current knowledge and attitude towards smoking. Gender-specific approaches to smoking interventions need to be developed.

## Introduction

Over 1.1 billion people smoked cigarettes in 2015 and regardless of the anti-smoking international effort, the number of people who are continue to smoke is increasing globally [1]. Smoking is one of leading preventable causes of death. That is, each year six million people die as a direct result of cigarette smoking [1]. Smoking is also associated with cancer, cardiovascular problems, respiratory problems and stroke [2]. The magnitude of negative consequences on younger populations are more severe. While including the above mentioned health problems, smoking also impairs young peoples’ physical fitness in terms of performance and stamina and increase their likelihood of substance dependency and abuse [3]. Smoking is also linked to the earlier onset of mental illness and higher rates of smoking are also found in people who have a mental illness which leads to significant co-morbidities [4].

Oman is a developed county in Southwest Asia with a total population of 4.5 million people and almost half are expatriates [5]. The health care system of Oman is developed and it has improved significantly since 1970 as extraordinary efforts have been exerted to improve the general health of the Omani population. However, smoking has received less attention compared with other health problems such cardiovascular disease, diabetes and cancer [6] That is, although smoking is one of leading causes of death and one which is linked to the development of many diseases, the actual magnitude of the problem is not clear in Oman. The last national survey on smoking conducted in Oman was in 2004. The results showed that around 7% of Omani population were cigarette smokers [7]. However, the percentage has increased to 11% of total Omani population in 2015 and this rate is predicted to double by 2025, though the report did not comment on the likely reasons (WHO), [8]. The WHO alludes to the importance of conducting a national survey every 5 years to ascertain rates of smoking in young adults but in 2015 there was no any national surveys conducted (WHO), [8]. This research aimed to redress this issue and respond to WHO call to conduct ongoing surveys in this area.

One method of targeting young adults is to identify the prevalence of smoking among university students. Previous studies showed that the prevalence of smoking among university students varied across countries. In Kuwait 42% of total university students self-reported that they were smokers [9]. In Saudi Arabia, the prevalence ranged from 30 to 35% among male students [10, 11]. Another study from Jordan showed similar results to previous studies conducted in Saudi Arabia [12]. Given the percentage of university students who are smokers, exploring the knowledge and attitude toward smoking to this age group is required.

Commencing university is a transition time when many young adults move from the family home to more independent lifestyles and liberal environments. In this critical transition life period, students may be exposed for the first time or experience peer pressure to start new social experiences such as smoking. However, there are no current epidemiological studies that explore the magnitude of the problem between Omani university students as clearly stated in WHO last report. Only the prevalence of smoking among adult population. In fact, WHO recommends clearly conducting study on youth population.

### Aim of Study

The aim of this research was to: (a) Assess the prevalence of smoking and associated sociodemographic and economic factors among university students in Oman; and, (b) Examine students’ knowledge about and attitudes towards smoking.

## Methods

### Design

A cross-sectional descriptive design was utilized as this design is useful to generate a snapshot of information in a short period of time.

The research was approved by the Research Ethics Committees at Sultan Qaboos University (SQU), Nizwa University (NU), and the University of Buraimi (UoB) (REC/2017-2018/10). The inclusion criteria for the research were Omani students registered at one of the above universities who provided informed consent to participate in the research. Following ethics approval, random lists of students and their contact information were generated by the Deanship of Admission and Registration at each university. An email including general information about the study, an informed consent statement and the contact information of the research assistants (RAs) was sent to students by the Public Relations and Information Department in respective universities.

Students who were interested in participating contacted the RAs who answered questions the students had and organized a convenient meeting time on campus for the student to complete the surveys. Prior to completing the surveys participants were provided with an information sheet and signed a consent form. When participants completed the survey, they put it in a sealed envelope and placed in in the survey box. No identifying information was collected and data will be presented in aggregate to maintain confidentiality.

### Setting

Sultan Qaboos University (SQU), Nizwa University (NU), and the University of Buraimi (UoB) are in different geographical areas of Oman and students who attend the campuses come from all over Oman they were chosen to ensure the heterogeneity of the sample.

SQU is the only governmental university, located in the capital city of Oman and established in 1986 has around 16,000 students. NU is a non-profit private university established in 2005 located in Nizwa, Ad Dakhiliyah region has approximately 6000 students [13]. UoB is a private university established in 2004 located in the Al Buraimi Governorate in the Northern part of Oman with 2100 students [13].

### Sample

Proportionate random sampling technique was used to recruit participants in the study. The total number of students at the SQU; NU; and UoB was approximately 24,100 students. In order to calculate the sample size, Sloven’s formula was used n = N/(1 + N e2), where n = Number of samples, N = Total population and e = margin of error, (0.05) which resulted in an n = 393.

A random sample of 400 students were used for this study. A random sample of all students in SQU, NU, and UoB who met the inclusion criteria and agreed to take part in the study were included. To ensure representativeness a 66%, 25%, and 9% of students at SQU, NU, and UoB was chosen from each respective university. Therefore, 264 students were randomly selected from SQU; 100 students were randomly selected from NU; and 36 students were randomly selected from UoB.

### Questionnaire and Measures

The research team member designed and developed a new survey based on the previous literature and well-known global surveys, which are available in public domains, the Global Adult Tobacco Survey (GATS) version 2.1 [14], Global Youth Tobacco Survey (GYTS) [15], California Adult Tobacco Survey [16], and Youth Tobacco Survey (YTS) [17].

The survey included information about (1) participants’ demographics and past history of smoking; (2) knowledge survey, and (3) positive attitudes towards smoking survey.

The knowledge survey consisted of 16 items with Yes or No answers. The correct answer received one mark and wrong answer received zero mark. Higher scores meant higher knowledge about smoking. Examples of knowledge questions were “smoking increases the risk of cardiovascular problems” and “smoking increases the risk of having a premature baby”. The maximum score that could be obtained was 16.

The attitude survey contained 12 positive statements about smoking with Yes No response, for example, “smoking increases self-esteem”, “people who smoke are more attractive”, and “smoking helps you to relax”. For each statement answered yes, one mark was given to the participants. Higher score meant more positive attitude toward smoking. The maximum score that could be obtained was 12.

One Professor of Public Health and three Nursing Professors to obtain face reviewed the developed survey to obtain face and content validity to ensure the survey was free of typographical errors. Reliability tests were conducted for both of the knowledge and attitude scales. Cronbach’s alpha was 0.818 for the16-items’ knowledge survey and 0.896 for the 12-items’ attitude survey.

### Analysis

Data were entered into SPSS (version 20) program for the analysis (SPSS, Chicago, IL, USA). Descriptive statistics were used such as frequency, means, and standard deviations to describe sample characteristics and other factors. Inferential statistics used included Independent t-test, and Chi-square to draw conclusions about variables under examination.

The difference between means of age, knowledge score, attitude score, years spent in the university, student income/day, and family members who are smoking were analysed using t-test. The remaining factors such as gender, marital status, location, college, father’s level of education and mother’s level of education were analysed using Chi-square. A p-value ≤ 0.05 was considered to be statistically significant.

## Results

Four hundred and one students completed the survey. The mean age of participants was 21.6 (SD = 2.0) years. Around two third of students were males 235 (58.6%) and the majority were single 378 (94.3%). One third of the students were from College of Art and Social science 123 (30.7%). The majority of students were not smokers 364 (90.8%). The prevalence rate of smoking was 9.0%. See Table 1 for more results.

**Table 1:** Sample characteristics (N = 401)

Variable	N	%	Variable	N	%
Gender			Marital status		
Male	235	58.6	Single	378	94.3
Female			Married	23	5.7
Father’s level of education			Mother’s level of education		
Illiterate	38	9.5	Illiterate	75	18.7
Elementary	41	10.2	Elementary	60	15
Secondary	149	37.2	Secondary	155	38.7
Diploma	45	11.2	Diploma	35	8.7
University	128	31.9	University	76	19
In the last 12 months, did you try stop smoking			In the last 12 months, did you receive any medical advice to stop smoking		
Yes	36	9	Yes	44	11
No	365	91	No	356	88
Smoking should be banned in public places			Anti-smoking campaign are required		
Yes	372	92.8	Yes	290	72.3
No	29	7.2	No	111	27.7
Factors encourage smoking—Multiple responses (n = 443)			Factors inhibit smoking multiple responses (n = 280)		
Curiosity	29	6.5	Negative physical health	114	40.7
Maturity	3	0.7	Religious	60	21.4
Peer pressure	8	1.8	Unhealthy routine	32	11.4
Pleasure	9	2	Saving money	14	5
Charming	3	0.7	My family hate smoking	14	5
Freedom	1	0.2	Unacceptable in community	21	7.5
Life pressure	11	2.5	Parents fear	12	4.3
Study pressure	12	2.7	I do not smoke	13	4.6
Family problems	2	0.5	Variable	M	SD
I do not smoke	364	82.2	Age	21.43	1.6
People who knows that you are smoking			Years spent in university	4	1.3
Family	2	0.5	Family income	1191	804
Friends	19	4.7	Family members who are smoking	2.29	1.5
Family and friends	7	1.7	Age when you start smoking	17.94	2.95
No one	7	1.7	Student income/day	4.24	3.2
I do not smoke	364	91			

*Omani riyals

Independent t-test results revealed a significant difference in years spent in the university, student income/day, family members who were smokers, knowledge score and attitude score. Students who were non-smokers had higher knowledge scores and a less positive attitude towards smoking scores. Table 2 details these results.

**Table 2:** Independent t-Test Results of students’ characteristics and smoking

	Smoking status, m (SD)				
Variable	Yes	No	t	df	*p*-value
Age	22 (1.26)	21.4 (1.6)	2.23	398	0.026*
Years spent in university	4.4 (1.04)	3.7 (1.45)	2.66	392	0.008*
Average family income	1541(1101)	1129 (698)	2.82	337	0.005*
Student income/day	6.36 (2.48)	4.16 (3.10)	4.1	392	< 0.001*
cGPA	2.69 (0.59)	2.81 (0.59)	− 1.06	291	0.289
Family members who are smokers	2.25 (1.55)	0.28 (0.82)	12.34	396	< 0.001*
Knowdge	11.97(3.69)	13.9(2.48)	− 4.23	398	< 0.001*
Positive attitudes	6.58(3.36)	3.65(3.71)	4.56	398	< 0.001*

^*^p < 0.05

Chi-square analyses were used to compare smokers and non-smokers in relation to the students’ characteristics including gender, marital status, location, college, father’s level of education and mother’s level of education. A significant difference was found in relation to gender, place of living, and if they had received medical advice (Table 3).

**Table 3:** Chi-square results of students’ characteristics

Variable	Smoker n (%)	Non-smoker n (%)	Chi square tests
Gender			
Male	30 (12.8)	204 (86.8)	
Female	6 (3.6)	160 (96.4)	X^2^ (2, N = 401) = 10.76 P = 0.005
Marital status			
Single	34 (9.0)	343 (90.7)	
Married	2 (8.7)	21 (91.3)	X^2^ (2, *N* = 401) = .064 P = 0.969
Place of living			
In-campus	5 (3.6)	135 (96.4)	
Out-campus	31 (11.9)	229 (87.7)	X^2^ (2, *N* = 401) = 8.29 P = 0.016
Father’s level of education			
Illiterate	3 (7.9)	35 (92.1)	
Elementary	1 (2.4)	39 (95.1)	
Secondary	11 (7.4)	138 (92.6)	X^2^ (8, *N* = 401) = 13.51
Diploma	5 (11.1)	40 (88.9)	
University	16 (12.5)	112(87.5)	P = 0.095
Mother’s level of education			
Illiterate	12 (16.0)	63 (84.0)	
Elementary	3 (5.0)	57 (95.0)	
Secondary	8 (5.2)	146 (94.2)	X^2^ (8, *N* = 401) = 13.22
Diploma	2 (5.7)	33 (94.3)	P = 0.104
University	11 (14.5)	65 (85.5)	
In the last 12 months, did you receive any medical advice to stop smoking			
Yes	10(22.7)	34(77.3)	X^2^ (4, *N* = 401) = 11.59
No	26(7.3)	329(92.4)	P = 0.021

## Discussion

The aim of this study was to assess the prevalence of smoking, associated factors, knowledge and attitudes towards smoking among university students in Oman. Our study revealed that 9.0% of the students were smokers which is a smaller proportion compared to similar populations in other neighbouring countries. In Saudi Arabia, between 16% [18] and 17% [19] of university students were smokers, in Bahrain 13% were daily smokers [20], in United Arab Emirates 15%, and in Kuwait 46% were reported to be smokers [21]. When comparing the results of the current study with the previous studies conducted in Oman among university students, the prevalence was slightly reduced from 10.1% [22]. The results of the current study were expected in Omani context because the Ministry of Health in Oman in collaboration with WHO, has created a national health plan with several interventional strategies to reduce the incidence of smoking among the Omani people (WHO [23]). This national plan includes a comprehensive smoking control program, increasing taxes on smoking, and banning of smoking cigarettes in enclosed public places (WHO [24]). In addition, in 2019 Oman also hosted a workshop on implementing tobacco taxes in countries of the WHO's Eastern Mediterranean Region to discuss and agree upon a way forward to implement national tobacco taxation policies.

Universities are complying with the public regulations of banning smoking in buildings and public places. However, universities need to promote smoking cessation clinics, initiate antismoking campaigns, and start health promoting anti-smoking clinics. In addition, universities and in their health elective courses need to increase the awareness of students about the negative health consequences of smoking.

Interestingly, our results showed that most of the students held negative attitudes towards smoking and had a good level of knowledge about smoking which might also explain the low rate of smokers in our sample. Positive attitudes towards smoking have been shown to be associated with commencing tobacco smoking in a longitudinal study among university students [25]. However, contrary to our findings, the same study could not find a significant association between the students’ knowledge level and the commencement of smoking. Therefore, further longitudinal studies in Oman could add to the existing body of knowledge.

A study conducted among university students also found that smokers had negative attitudes towards policy aimed to reduce students smoking, such as banning of smoking in public areas on campus [26]. Musmar [26] showed further that smokers were less likely to acknowledge education about the harmful effects of smoking. These findings taken together with the results from current study strengthen the need for health promoting and strengthening campaigns to sustain and increase the existing negative attitudes towards smoking in Omani university students. In addition, a clear action plan needs to be developed by the healthcare authorities and /or public health sector to continue to educate and change attitudes to reduce levels of smoking.

Another interesting finding reported in this study was the significant differences between smokers and non-smokers regarding student income per day and family members who are smoking. Similar findings were reported in a qualitative study addressing the factors that motivate male university students to smoke in Kuwait [27]. The study addressed family, social, and psychological factors to be motivational to smoke [27]. Thus, an effective and sustainable health education program should be planned to take into consideration the possible associated factors with smoking initiation and positive attitudes towards smoking. In addition, policy makers in Ministry of health and public health sector in collaboration with university administration should introduce a clear smoking prevention plan. Such a program should be introduced at a younger age to promote healthy living.

Additionally, gender was one of the factors associated with smoking status in the current study. Compared to studies conducted in similar populations, i.e. university students in the Middle East, the gender differences in our study are in line with the smoking trends in the neighboring countries. In Musmar [26], the rate of male students who were smoking was significantly higher compared to their female counterparts. Furthermore, in a review study about the prevalence of smoking in university students, the authors found that the smoking prevalence among male students was significantly higher than female students in several Arab countries such as Saudi Arabia, Bahrain, Yemen, Jordan, Egypt, Tunisia, and Palestine [21]. The significant differences in smoking among males and females could be explained in the light of the nature of the Middle Eastern region with its customs and traditions according to which smoking among females is still culturally unacceptable. This calls for gender-specific approaches to smoking interventions among Omani university students.

The findings of the current study give a general background information and emphasis on the need for decision makers in Oman to develop new strategies and educational programs that help in reducing the prevalence of smoking among university students.

### Limitation

A limitation of this study is the nature of the descriptive point prevalence study that measures the actual prevalence in a certain point in time but does not explain causes or relations, i.e. only associations but not the direction of the association. However, this type of study is needed to generate hypotheses, construct research questions, and guide policy makers as well as researchers in further steps.

## Conclusions

A low rate of smokers in this population is a good sign of the effectiveness of the public health policies followed in Oman. However, predictions suggest this trend of low rates of smoking may increase due to the influences of peers, family members, and the psychological factors. Thus, strategies that promote a sustainable healthy living need to be developed and implemented in younger populations, especially university students. Additionally, gender was one of the factors associated with smoking status, which calls for gender-specific approaches to smoking interventions among Omani university students. Further research with a qualitative approach to investigate smoking initiation could be of significant importance to support the current study findings and give a more nuanced picture of the smoking habits among university students.
